# Targeted Approaches to Inhibit Sialylation of Multiple Myeloma in the Bone Marrow Microenvironment

**DOI:** 10.3389/fbioe.2019.00252

**Published:** 2019-10-04

**Authors:** Alessandro Natoni, Raghvendra Bohara, Abhay Pandit, Michael O'Dwyer

**Affiliations:** ^1^Apoptosis Research Centre, School of Medicine, National University of Ireland, Galway, Ireland; ^2^Centre for Research in Medical Devices (CÚRAM), National University of Ireland, Galway, Ireland

**Keywords:** multiple myeloma, microenvironment, sialylation, targeted delivery, chemotherapy, ST3GAL6, E-selectin, integrins

## Abstract

Aberrant glycosylation modulates different aspects of tumor biology, and it has long been recognized as a hallmark of cancer. Among the different forms of glycosylation, sialylation, the addition of sialic acid to underlying oligosaccharides, is often dysregulated in cancer. Increased expression of sialylated glycans has been observed in many types of cancer, including multiple myeloma, and often correlates with aggressive metastatic behavior. Myeloma, a cancer of plasma cells, develops in the bone marrow, and colonizes multiple sites of the skeleton including the skull. In myeloma, the bone marrow represents an essential niche where the malignant cells are nurtured by the microenvironment and protected from chemotherapy. Here, we discuss the role of hypersialylation in the metastatic process focusing on multiple myeloma. In particular, we examine how increased sialylation modulates homing of malignant plasma cells into the bone marrow by regulating the activity of molecules important in bone marrow cellular trafficking including selectins and integrins. We also propose that inhibiting sialylation may represent a new therapeutic strategy to overcome bone marrow-mediated chemotherapy resistance and describe different targeted approaches to specifically deliver sialylation inhibitors to the bone marrow microenvironment.

## Introduction

The development and progression of human malignancies from the first appearance of benign lesions to the emergence of deadly metastatic clones consist of a multi-step process involving the progressive acquisition of selective alterations that ultimately result in the pathological behavior of cancer cells. Glycosylation represents one of these processes that are often dysregulated in cancer. It is one of the most important co- and post-translational modifications that contributes to and modulates the many diverse functions of glycoconjugates. This extraordinary range of biological functions originates from the complexity and the diversity found within the glycan structure such as carbohydrate composition and linkage, anomeric state, branching, presence of additional modification (sulfation), and linkage to their aglycone part (carrier). As the majority of known glycoconjugates are exposed to the outer surface of the plasma membrane or secreted into the extracellular milieu, they mediate important biological processes such as cell adhesion, migration, immune response, as well as receptor activation and intracellular signaling. During tumor development, the glycan profile undergoes a profound and dynamic alteration that allows cancer cells to acquire novel traits necessary for tumor progression. These glycosylation changes are not random and involve, among others, *de novo* synthesis of new carbohydrate structures, premature termination of pre-existing glycans, and increased expression of terminal sialylated glycans. Acquisition of sialylated structures represents one of the most important modifications of the glycome during tumor development, and it is often associated with an aggressive metastatic phenotype. However, the study of the role of sialylation in cancer is still in its infancy and strategies to efficiently and safely target this important biological process are still lacking.

## Multiple Myeloma: a Metastatic Disease that depends on the Bone Marrow Microenvironment

Multiple myeloma (MM) arises from clonal expansion of terminally differentiated plasma cells in the bone marrow (BM). MM is usually preceded by asymptomatic precursor states called Monoclonal Gammopathy of Undetermined Significance (MGUS) and Smoldering MM (SMM). Genetic abnormalities, epigenetic alterations, and microenvironmental factors co-operate in the development of symptomatic MM (Bianchi and Munshi, 2015). The BM microenvironment represents the perfect niche where MM cells proliferate and become resistant to chemotherapeutic drugs (Manier et al., 2012). A combination of soluble growth factors and adhesion molecules mediate these pro-survival and proliferative signaling pathways (Di Marzo et al., 2016). This extreme dependency on the BM suggests that malignant cells could be particularly vulnerable in the circulation where the effective concentration of a chemotherapeutic drug is higher than in the BM and where they are more susceptible to an immune response. Thus, MM cells must have evolved strategies to enhance their survival in the bloodstream such as mechanisms of immune evasion and efficient homing into the BM. Supporting this hypothesis is the evidence that MM is highly metastatic, colonizing different sites of the axial skeleton including the skull (Moschetta et al., 2017). Homing of MM cells into the BM is primarily mediated by stromal cell-derived factor 1α (SDF1α) and its receptor C-X-C chemokine receptor type 4 (CXCR4) (Alsayed et al., 2007). This chemokine also plays a role in adhesion and possibly retention of MM in the BM via α4β1-dependent adhesion on fibronectin and vascular cell adhesion molecule 1 (VCAM-1) (Gazitt and Akay, 2004; Parmo-Cabanas et al., 2004; Menu et al., 2006). Besides SDF1α, other molecules have been shown to be important in homing and adhesion of MM to the BM. These include integrin α4β1, α4/β7, and P-selectin glycoprotein ligand-1 (PSGL-1), all of which are highly expressed on MM cells (Sanz-Rodriguez et al., 1999; Florena et al., 2005; Neri et al., 2011). Notably, these molecules, including SDF1α, are also involved in cell adhesion-mediated drug resistance (CAM-DR) and therefore represent attractive targets for MM therapy (Damiano et al., 1999; Azab et al., 2009; Muz et al., 2015; Waldschmidt et al., 2017). Although these molecules have been shown to be important in regulating critical biological processes involved in the progression and development of MM, little is known about how post-translational modifications influence their functions. Above all, the role of sialylation in regulating some of the biological functions of these molecules has only been recently recognized. Secretion of extracellular vesicles (EVs) by malignant plasma cells represents another important mechanism of MM dissemination (Colombo et al., 2019). Indeed, MM-EVs have been found both in MM patients' peripheral blood (PB) and BM, and their levels in bloodstream positively correlate with the number of bone lesions (Zhang L. et al., 2019). It has been proposed that EVs have an important role in different steps of the metastatic process (Colombo et al., 2019). Due to their pro-coagulant activity, EVs could lead to platelet activation and polymerization of fibrinogen to fibrin, which in turn would enhance MM dissemination by protecting the malignant plasma cells in the circulation, favoring their seeding to distant sites and pre-conditioning the metastatic niche with platelet-derived cytokines (Labelle et al., 2014; Remiker and Palumbo, 2018; Nielsen et al., 2019). It has also been shown that MM-EVs contribute to neo-angiogenesis by inducing endothelial cell proliferation and formation of new blood vessels (Liu et al., 2014; Wang et al., 2016; Li et al., 2019; Zarfati et al., 2019). Whether MM-EV sialylation status is important for some or all their biological functions is yet to be confirmed. It is also tempting to speculate that MM-EVs could also alter the sialylation status of the metastatic niche favoring MM seeding and homing into the BM. Although this is just a hypothesis, it represents an exciting field for future studies.

## Sialylation: an Important co- and Post-Translational Modification in Cancer Biology

Sialic acid refers to a group of ~50 chemical variants of the nine carbon sugar neuraminic acid that are strategically placed at the tip of glycans. At physiological pH, sialic acids confer a negative charge to the underlying glycoproteins and glycolipids, which contribute to their physiological and biophysical functions. Indeed, sialic acids provide charge repulsion on human erythrocytes and other cells, preventing unwanted interactions of cells in the circulation. In the kidneys, sialic acids are critical in maintaining their normal filtering function (Gelberg et al., 1996), and extended polysialic acid chains can affect neuronal plasticity (Rutishauser, 2008). Moreover, sialic acids represent critical determinants of selectin ligands, which contribute to extravasation of immune cells to target organs and sites (Mcever, 2015). Pathologically, sialic acids are involved in many infectious diseases, being the targets for the binding of a large number of pathogenic organisms and their toxins, and in cancer (Varki, 2008). Indeed, aberrant sialylation is observed in many cancers and it seems to be a major contributor of the metastatic phenotype (Schultz et al., 2012).

Sialic acids can be added to the underlying sugars through different linkages: an α2-3- or an α2-6-bond to galactose (Gal); an α2-6-bond to N-acetylgalactosamine (GalNAc) or N-acetylglucosamine (GlcNAc); and an α2-8-bond to another sialic acid, forming polysialic acid. These linkages are elaborated by a family of 20 sialyltransferases (STs), a class of glycosyltransferases that share the cytosine monophosphate (CMP)–sialic acid as substrate donor, but differ in tissue distribution, in the catalysis of the specific glycosidic linkage and in the recognition of the acceptor oligosaccharide and/or glycoconjugates (Harduin-Lepers et al., 2001). All the STs are highly stereoselective and catalyze the formation of an α-linked sialic acid to a precise hydroxyl group on a specific saccharide residue. The combination of the acceptor saccharide residue and the precise hydroxyl group on this residue can be used to divide the family into four distinct sub-families. For example, ST6Gal enzymes catalyze the transfer of sialic acid to the 6′-hydroxyl group of a Gal residue, ST3Gal enzymes catalyze the transfer of sialic acid to the 3′-hydroxyl group of a Gal residue, ST3GalNAc enzymes catalyze the transfer of sialic acid to the 6′-hydroxyl group of a GalNac, and ST3Sia enzymes catalyze the transfer of sialic acid to the 8′-hydroxyl group of another sialic acid residue. The number of the STs exceeds by far the number of existing sialyl linkages, suggesting a certain degree of redundancy. However, although there are six STs capable of catalyzing the transfer of sialic acid to an underlying Gal residue in an α2-3 linkage, only two of them, namely, ST3GAL4 and ST3GAL6, have been implicated in synthetizing selectin ligands (Rodrigues and Macauley, 2018). Thus, it might be possible that the structure of the underlying glycans together with the protein or lipid backbone dictate the specificity of the different STs.

Aberrant sialylation can arise as a result of different mechanisms including overexpression of STs, dysfunction of the enzymes that controls the synthesis and availability of CPM–sialic acids and downregulation of sialidase, the enzymes that cleave off sialic acids from glycans (Rodrigues and Macauley, 2018). Aberrant sialylation seems to be particularly important in promoting tumor dissemination, survival in the circulation, adhesion on the target endothelium, and extravasation (Schultz et al., 2012). For example, ST6GAL1 has been implicated in many metastatic tumors (Garnham et al., 2019). Physiologically, ST6GAL1 is involved in the generation of CD22 ligands that control B cell functions (Clark and Giltiay, 2018). However, upregulation of ST6GAL1 in cancer leads to enhanced migration and invasiveness by specifically inducing sialylation of the integrin β1 (Seales et al., 2005; Christie et al., 2008; Shaikh et al., 2008). Selectin ligands represent another class of molecules that are regulated by sialylation and are instrumental in directing tumor cell adhesion on the endothelium of target organs during metastatic dissemination (Natoni et al., 2016). Sialyl Lewis (sLe) structures are essential determinants for the function of selectin ligands. These are tetrasaccharide structures composed of a GlcNAc-Gal backbone, an α2-3-linked sialic acid to Gal, and a fucose linked to GlcNAc either with an α1-3 (sLe^x^) or an α1-4 linkage (sLe^a^). Through selectin ligand/selectin interactions, tumor cells are able to tether and roll on the vascular endothelium in a process that closely mirror leukocyte extravasation during inflammation or homing (Gout et al., 2008). This represents the first step in cancer seeding and colonization of target organs. Notably, the expression of selectin ligands on cancer cells seems to correlate with metastatic phenotype (Fukuoka et al., 1998; Tatsumi et al., 1998; Ben-David et al., 2008; Geng et al., 2012; Li et al., 2013) and disease progression (Chien et al., 2013; Gakhar et al., 2013) and negatively correlate with patient survival (Amado et al., 1998; Baldus et al., 1998; Grabowski et al., 2000; Woodman et al., 2016).

Aberrant sialylation can indirectly promote metastatic spread by increasing survival of metastatic cells in the circulation. P-selectin ligands on tumor cells interact with P-selectin on platelets, inducing the formation of a platelet cloak that efficiently suppresses the innate immune system and promotes metastasis (Placke et al., 2012; Cluxton et al., 2019). Sialoglycans are also recognized by a class of immunomodulatory receptors named sialic acid-binding immunoglobulin-type lectins (Siglecs), which mainly serve as negative modulators of the immune system (Rodrigues and Macauley, 2018). In this respect, sialoglycans can be considered as self-associated molecular patterns (SAMPs) that can lead to inhibition of immune responses against self. Hypersialylation can also induce immune evasion by interference with the complement system via a “molecular cloaking” mechanism mediated by Factor H sequestration and dampening of the complement-mediated cell lysis and opsonization (Fedarko et al., 2000). Thus, hypersialylation can contribute to the metastatic phenotype of cancer cells with a variety of mechanisms that interfere with different biological processes spanning from immune surveillance to adhesion.

## Role of Sialylation in the Biology of Myeloma

The first evidence of altered sialylation in MM dates back to 1984, when an increase in ST activity was reported in mononuclear cells and serum derived from the PB and BM of MM patients compared to MGUS and healthy individuals (Cohen et al., 1989). However, despite this important observation, there was no functional characterization of this enhanced ST activity. Direct evidence of a role of aberrant sialylation in MM came from the observation that ST3GAL6, a ST responsible for the generation of α2-3-linked sialic acids, was highly expressed in MM and correlated with inferior overall survival (Glavey et al., 2014). In particular, it was shown that ST3GAL6 knock-down reduced MM adhesion on BM stromal cells and transendothelial migration *in vitro* and MM homing into the BM *in vivo*. This study revealed for the first time a functional link between sialylation and homing of MM into the BM and inspired subsequent studies aiming at understanding the underlying molecular mechanism(s). ST3GAL6 contributes to the generation of sialofucosylated structures such as Sle^a/x^ that are recognized by E-selectin (Yang et al., 2012) and may directly participate to the generation of E-selectin ligands on MM cells. Importantly, E-selectin is constitutively expressed on the BM endothelium (Schweitzer et al., 1996; Sipkins et al., 2005) and plays a crucial role in homing of human hematopoietic stem cells (HSC) into the BM (Hidalgo et al., 2002). It has also been shown that E-selectin drives cancer metastasis into the BM (Price et al., 2016; Esposito et al., 2019). Since MM metastasizes into the BM and expresses high levels of ST3GAL6, it is conceivable to hypothesize that E-selectin, together with SDF1α, plays an important role in MM homing. Indeed, it was recently shown that E-selectin ligands expressed on MM cells played a role in BM homing and possibly retention of the malignant cells in the BM (Martinez-Moreno et al., 2016; Natoni et al., 2017). Notably, it was shown that MM cells enriched for E-selectin ligands recognized by the monoclonal antibody Heca452 (MM^Heca452Enriched^) were resistant to bortezomib treatment *in vivo*, and this resistance was reversed by a small glycomimetic molecule, GMI-1271, which inhibits E-selectin/E-selectin ligand interaction (Natoni et al., 2017). The mechanism(s) of this bortezomib resistance displayed *in vivo* by the MM^Heca452Enriched^ cells is still not known. An intriguing hypothesis is that the presence of E-selectin ligands on the MM^Heca452Enriched^ cells facilitate their homing into the BM, which then provides protection to bortezomib-induced cell death. In this scenario, E-selectin ligands ensure efficient trafficking into the BM, decreasing the time MM^Heca452Enriched^ cells are exposed to bortezomib in the circulation. In line with this hypothesis, we found that GMI-1271 treatment induced an accumulation of MM^Heca452Enriched^ cells in the peripheral blood where they may be more sensitive to bortezomib. Alternatively, E-selectin may retain the MM^Heca452Enriched^ cells into the BM and GMI-1271 induced egress of these cells into the circulation, a mechanism that has been proposed for leukemic stem cells (Winkler et al., 2014). Obviously, a combination of both mechanisms could be possible.

In addition to the generation of selectin ligands, sialylation plays other roles in MM. Indeed, we have recently shown that the sialylation status of the α4 subunit of integrins α4β1 and α4β7 modulates their activity (Natoni et al., 2019). Specifically, we have shown that inhibition of sialylation using 3F_ax_-Neu5Ac, a global inhibitor of the ST family (Rillahan et al., 2012), impaired maturation of the α4 chain, greatly reducing the ability of MM cells to interact with VCAM-1 and mucosal vascular addressin cell adhesion molecule 1 (MADCAM-1). VCAM-1, the counter-receptor for integrin α4β1, is expressed by the BM endothelial and stromal cells (Simmons et al., 1992; Schweitzer et al., 1996) and participates in MM adhesion on BM endothelial cells (Okada et al., 1999; Parmo-Cabanas et al., 2004). MADCAM-1, the counter-receptor for integrin α4β7, is also expressed in the BM and promotes homing of HSCs into the BM (Katayama et al., 2004; Tada et al., 2008; Murakami et al., 2016). Similarly, MADCAM-1 together with E-selectin and SDF1α could facilitate homing of MM into the BM. Thus, global inhibition of sialylation may have a broad impact on MM homing. Indeed, we have shown that using a mouse model identical to our previous study, 3F_ax_-Neu5Ac not only improved survival by enhancing bortezomib sensitivity but also prolonged survival and reduced tumor burden even in the absence of bortezomib, indicating that inhibition of sialylation was beneficial even without the addition of chemotherapy (Natoni et al., 2019).

Sialylation seems to regulate the function of CD147, also known as extracellular matrix metalloproteinase inducer (EMMPRIN). CD147 has been shown to be involved in MM proliferation and metastasis (Zhu et al., 2015). Importantly, sialylation of CD147 is upregulated by IL-6 (Wang et al., 2017). This observation opens the fascinating possibility that sialylation is also regulated by the microenvironment. Indeed, in the same study, it was shown that IL-6 transcriptionally upregulated several STs, such as *ST3GAL3, ST3GAL6*, and *ST6GAL1* through the activation of signal transducer and activator of transcription 3 (STAT3) (Wang et al., 2017). It should be noted that ST6GAL1 has been proposed to be the main ST regulating the sialylation of the integrin subunit β1; thus, it might impact migration, adhesion, and dissemination of MM cells (Seales et al., 2005; Christie et al., 2008; Shaikh et al., 2008).

Another microenvironmental factor that regulates the expression of STs and, in particular, ST3GAL6 is hypoxia (Glavey et al., 2014). MM develops in the BM, which represents an extremely hypoxic niche (Hu et al., 2010), and it has been shown that hypoxia promotes MM dissemination (Azab et al., 2012). Thus, upregulation of STs may represent one of the mechanisms by which hypoxia facilitates metastatic spread of MM cells.

Besides the microenvironment, ST expression in MM can also be regulated by long non-coding RNAs (lncRNAs). Indeed, it has been recently shown that the mRNA levels of *ST3GAL6* are regulated by the lncRNA ST3GAL6-AS1 (Shen et al., 2018a; Vinci et al., 2018). Interestingly, ST3GAL6-AS1 is upregulated in MM, and its mRNA levels directly correlate with those of ST3GAL6 (Shen et al., 2018b; Vinci et al., 2018). Knocking down ST3GAL6-AS1 results in decreased migration, invasion, and adhesion of MM to fibronectin, HUVEC, and collagen type I (Shen et al., 2018a). Although the molecular mechanisms responsible for these phenotypes are still unknown, ST3GAL6-AS1 knock-down causes a decrease in ST3GAL6 levels, suggesting co-regulatory mechanism(s), resulting in impaired sialylation.

It has been recently shown that the monoclonal immunoglobulins secreted by the malignant plasma cells display an aberrant glycosylation pattern (Zhang Z. et al., 2019). In particular, a detailed analysis of the total protein *N*-glycans derived from serum samples of MM patients showed a decrease in the α2-3- and α2-6-linked sialic acids on secreted monoclonal immunoglobulins. Thus, it might be possible that alterations in the ST expression pattern in MM favor an increase in sialylation of the cell surface over secreted proteins, although this needs to be confirmed. Moreover, in the previous study, the *N*-glycans, but not *O*-glycans, were analyzed; thus, a more comprehensive view of the sialylation status of MM secreted proteins is still lacking.

As all these studies point out, aberrant sialylation plays an important role in MM, especially in the metastatic spread of the malignant cells and in their retention into the BM niche. The relevance of sialylation in MM is reinforced by the observation that the expression of ST genes such as *ST3GAL6* and *ST3GAL1*, either alone or in combination with other glycogenes, can identify patients with poor outcome (Glavey et al., 2013; Connolly et al., 2016). While ST3GAL6 has been implicated in the generation of E-selectin ligands (Yang et al., 2012), ST3GAL1 acts predominantly on Core-1 O-glycans, masking the tumor from surrounding immune cells (Burchell et al., 1999). Therefore, sialylation may well-represent an important target for future therapeutic strategies especially in combination with standard and novel chemotherapeutic drugs. However, a major obstacle to clinical development of current ST inhibitors is their potential to induce nephrotoxicity, which appears to be the major dose-limiting toxicity in *in vivo* studies (Macauley et al., 2014). This is particularly relevant in MM where renal impairment is common. While novel and more specific ST inhibitors may overcome this problem, targeted delivery systems capable of selectively releasing ST inhibitors into the BM microenvironment or the MM cells, could represent an attractive alternative solution. Besides nephrotoxicity, other potential side effects of global sialylation inhibition are those related to the hematopoietic system. Since sialic acid is an important determinant of E-, P-, and L-selectin ligands present on the immune cells (Varki, 1994; Sperandio, 2006; Zarbock et al., 2011), global sialylation inhibition will inevitably affect their biological functions, in particular leukocyte trafficking and extravasation. Elevated levels of α2-3-linked sialic acid together with overexpression of ST3GAL6 have been found in CD133 positive hematopoietic stem and progenitor cells (Hemmoranta et al., 2007). Moreover, it has been shown that expression of E-selectin in the BM is required for HSC homing into the BM (Hidalgo et al., 2002). Thus, sialylation inhibition would probably lead to hematopoietic stem and progenitor cell mobilization and impair their homing into the BM. However, it should be noted that E-selectin inhibition using the specific E-selectin inhibitor GMI1271 in clinical trials in MM (NCT02811822) and AML (NCT02306291) has not been associated with myelosuppression or other side effects.

## Liposome-Based Targeted Delivery in MM

Nanomedicine approaches offer the potential of enhanced and more personalized therapeutics in MM with a reduced risk of off-target effects (Table 1). Current treatments in MM rely on immunomodulatory drugs such as lenalidomide combined with proteasome inhibitors (PI) such as bortezomib (De La Puente and Azab, 2017). The introduction of PI inhibitors has contributed to major improvements in patient survival, but off-target effects, in particular neurotoxicity, remain a major concern (Richardson et al., 2009). Moreover, the emergence of drug resistance and how to deal with this has become a burning issue of research in MM. The tumor physiology offers a wide range of targeting opportunity, which can be designed or tuned and simultaneously co-delivered with chemotherapeutics for more personalized therapy in MM. Several groups have developed different systems to facilitate targeted delivery of chemotherapeutics in MM (Du et al., 2012). Targeting tumor physiology using antibodies, functional groups, or small molecules seems to be a more rational approach to minimize off-target effects. Kiziltepe et al. have shown that delivering doxorubicin using a lipid-PEG block polymer nano-formulation tagged by the VLA-4 antigen was significantly more cytotoxic to MM cells compared to the free drug (Kiziltepe et al., 2012). Using an alternative approach, Chang et al. developed dioleoyl phosphatidic acid (DOPA)-based paclitaxel (PTX)-loaded liposomes with modifications of alendronate and transferrin, which were able to target the bone microenvironment (Chang et al., 2016). Targeting the CD38 receptor in MM represents an additional attractive approach as CD38 is highly expressed on malignant plasma cells (De La Puente et al., 2018). Azab's group successfully prepared a chitosan nanoparticle formulation tagged with anti-CD38 monoclonal antibodies to deliver bortezomib *in vivo*, improving its therapeutic efficacy and reducing side effects (De La Puente et al., 2018). This formulation showed increased therapeutic activity and reduced off-target effects compared to the free drug. Thus, targeted delivery of chemotherapeutic drugs has proven valuable in MM, and a similar approach could be used to safely deliver 3F_ax_-Neu5Ac, avoiding kidney toxicity. Indeed, Büll et al. have shown that encapsulation of 3F_ax_-Neu5Ac in poly(lactic-co-glycolic acid) (PLGA)-based nanoparticles (NPs) targeting the melanoma antigen tyrosinase related protein-1 (TRP-1) allowed specific and prolonged blockade of sialic acid expression *in vitro* and precluded metastatic spread *in vivo* (Bull et al., 2015). A similar strategy could be employed using antibodies specific to MM antigens (such as CD38 and BCMA) or by incorporating bisphosphonates into the nanoparticles to target the BM (Swami et al., 2014). Inhibiting sialylation using these approaches could have also beneficial effects on the tumor microenvironment. For instance, it has been recently reported that sialic acid blockade via intra-tumoral injection of 3F_ax_-Neu5Ac could suppress tumor growth by enhancing T cell-mediated tumor immunity (Bull et al., 2018). Indeed, ST inhibition increased the number of activated effector immune cells, including CD8^+^ T cells and natural killer (NK) cells, along with a reduction in regulatory T cells (Tregs) and activation of dendritic cells (DCs).

**Table 1:** Liposome-based targeted treatment available for MM.

**References**	**Nanoparticles**	**Drug loaded**	**Targeting moiety**	***In vitro* study**	***In vivo* study**
Ashley et al., 2014	Liposomes	Carfilzomib	VLA-4 targeted	MM1S and NCI-H929 cell	SCID mice injected with NCI-H929 tumors
Braham et al., 2018	Liposomes	Doxorubicin Bortezomib	VLA-4 targeted	BM myeloma multipotent mesenchymal stromal cells, endothelial progenitor cells, and L363, and MM1S cells co-cultured in hydrogel.	N/A
Chang et al., 2016	Liposomes	Paclitaxel	Alendronate and transferrin	MM1S	MM1S GFP^+^ cells were injected into each mouse via the tail vein to prepare the tumor-bearing model
Lopes De Menezes et al., 2000	Liposomes	Doxorubicin	Anti-CD 19	Heterogeneous mixture of PBMC from MM patients and ARH77 cell	N/A
Maillard et al., 2005	Liposome	Hydroxy-tamoxifen (4-HT) or RU 58668	N/A	RPMI 8226	RPMI 8226 in female nude mice
Swami et al., 2014	PEG PLGA	Bortezomib	Alendronate	MM1S	NOD/SCID mice injected with MM1S GFP^+^ Luc^+^ cells
De La Puente et al., 2018	Chitosan	Bortezomib	anti-CD38	MM1S, RPMI 8226, NCI-H929, and U266	MM1S GFP^+^ Luc^+^-injected SCID mice
Yang et al., 2015	Fe_3_O_4_	Paclitaxel	Monoclonal antibody against ABCG2	RPMI 8266 cells and BM mononuclear cells	MM CSCs from human MM RPMI 8226 cells based on the CD138^−^CD34^−^cell phenotypes injected in NOD/SCID
Kotagiri et al., 2018	Nano micelles	Titanocene	VLA-4 targeted	MM1S	MM1S Luc^+^ cells in SCID mice

*ABCG2, ATP binding cassette subfamily g member 2; BM, bone marrow; CSCs, cancer stem cells; HA-P(TMC-co-DTC), HA-b-poly(trimethylene carbonate-co-dithiolane trimethylene carbonate) (HA-P[TMC-co-DTC]); GFP, green fluorescence protein; Luc, Luciferase; NOD, non-obese diabetic; MM, multiple myeloma; N/A, not available; PBMC, peripheral blood mononuclear cells; PEG, polyethylene glycol; PLGA, poly(lactic-co-glycolic acid); SCID, severe combined immunodeficiency; VLA, very late antigen*.

## Conclusion

Using different systems, it should be possible to safely target the delivery of 3F_ax_-Neu5Ac to the BM to inhibit sialylation in MM. We are currently working on a delivery system that employs 3F_ax_-Neu5Ac encapsulated into liposomes functionalized with bisphosphonates for selective delivery into the BM with promising results. This approach has the potential to block homing of MM into the BM and mobilize the malignant cells into the circulation, making them more sensitive to chemotherapeutic drugs. Moreover, release of 3F_ax_-Neu5Ac in the BM could have profound effects on the BM microenvironment. Indeed, we have recently shown that BM stromal cells cultured under conditions that mimic the inflammatory BM microenvironment in MM become highly immune suppressive and that this phenotype could be completely reverted by 3F_ax_-Neu5Ac (Lynch et al., 2017). Thus, sialylation is a valuable target in both MM cells and the BM microenvironment.

## Author Contributions

AN and RB conceived and wrote the manuscript. AP and MO'D proofed and approved the final version of the manuscript.

### Conflict of Interest

MO'D is listed as an inventor on the patent application US20170327899A1 https://patents.google.com/patent/US20170327899A1/en. The remaining authors declare that the research was conducted in the absence of any commercial or financial relationships that could be construed as a potential conflict of interest.
